# Illuminating the Intricacies: A Comparative Cross-Sectional Sonographic Evaluation of Degenerative Changes in Leiomyomas Through Post-Processing Technique

**DOI:** 10.3390/diagnostics15232943

**Published:** 2025-11-21

**Authors:** Mahasin G. Hassan, Nouf Aldrees, Sadeem Aldawsari, Raghad Alanazi, Noura Alboqami, Maryem Alanazi, Renad Alanazi, Khadejah Alrashidi, Basim S. Almutairi

**Affiliations:** 1 Department of Radiological Sciences, College of Health and Rehabilitation Sciences, Princess Nourah bint Abdulrahman University, P.O. Box 84428, Riyadh 11671, Saudi Arabia; 443000183@pnu.edu.sa (N.A.); 443000329@pnu.edu.sa (S.A.); 443000918@pnu.edu.sa (R.A.); 443000815@pnu.edu.sa (N.A.); 443000632@pnu.edu.sa (M.A.); 443000660@pnu.edu.sa (R.A.); 443000705@pnu.edu.sa (K.A.); 2 Department of MRI, Medical Imaging Administration, King Saud Medical City, Riyadh 12746, Saudi Arabia; b-almutairi@rfhc.gov.sa

**Keywords:** leiomyomas, degenerative changes, ultrasonography, Fiji, magnetic resonance imaging

## Abstract

**Background**: Leiomyomas are benign tumors that may cause symptoms and affect fertility, requiring careful assessment. Magnetic Resonance Imaging (MRI) becomes crucial when ultrasonography results are inconclusive; however, it is expensive and time-consuming. Utilizing post-processing techniques could enhance the ultrasound results. Using ultrasound with Fiji (ImageJ) enables precise evaluation of leiomyoma degeneration and may reduce the need for MRI. Aim: This study aims to evaluate the effectiveness of a post-processing technique using Fiji (ImageJ) to detect degenerative changes in leiomyomas and compare these findings with those obtained from conventional ultrasound and MRI results. **Methods**: A cross-sectional analytical study was conducted at King Saud Medical City involving 41 females diagnosed with uterine leiomyomas using ultrasound and MRI. Ultrasound images were analyzed using Fiji software to identify degenerative changes and compare results with ultrasound and MRI reports. **Results**: ImageJ outperformed ultrasound across all diagnostic metrics, with higher sensitivity (84.2% vs. 63.2%), specificity (81.8% vs. 22.7%), and accuracy (82.9% vs. 41.5%). ROC analysis showed superior diagnostic performance of ImageJ (AUC = 0.830) compared to ultrasound (AUC = 0.429), with a significant correlation to MRI findings (*p* < 0.001). Fibroids with and without degeneration showed no significant differences in Fiji parameters (*p* > 0.05). **Conclusions**: Integrating post-processing tools such as ImageJ with ultrasound imaging significantly improves the detection of degenerative changes in uterine leiomyomas, potentially reducing dependence on costly and less accessible modalities like MRI. Future studies should utilize a prospective design with larger sample sizes to strengthen the validity and generalizability of these findings.

## 1. Introduction

Leiomyomas, myomas, or fibroids, which are benign growths of muscle and connective tissue in the uterine wall, can grow large enough to distort the uterine surface or cavity. Although benign, they often cause severe symptoms, such as heavy, irregular, and prolonged menstrual bleeding [1,2] (Goad & Rajkovic, 2025; Sabry & Al-Hendy, 2012). Hormonal influences, particularly from estrogen and progesterone, significantly contribute to their growth; higher hormone levels seen during pregnancy and perimenopause increase the risk. Middle-aged individuals are at greater risk [3] (Constantini, 2020). Race (especially among African American women) is the strongest risk factor. Other factors include obesity, hypertension, vitamin D deficiency, non-parity, food additives, environmental exposures, and lifestyle factors [4] (Yang et al., 2022).

When a leiomyoma outgrows its vascular supply, it usually leads to tissue breakdown and inflammation, which causes pain as the body responds to the necrotic tissue (degeneration). Based on histopathological analysis, leiomyoma degeneration occurs in a variety of ways. About 60% of all degenerating leiomyomas undergo hyaline degeneration. Other forms include cystic (4%), calcification (4%), red (3%), and Myxoid (1–3%). In hyaline degeneration, the smooth muscle cells of the myoma are replaced by connective tissue. Cystic degeneration represents a more advanced stage, characterized by extensive edema and fluid-filled spaces. Myxoid degeneration involves the presence of cystic areas filled with gelatinous myxoid material situated between the smooth muscle fibers. Calcification is the final stage of degeneration and may indicate thrombosed veins due to previous deterioration [5,6] (Brown, 2011; McLucas, 2008). Because of their varied morphology and appearance, degenerating leiomyomas can be challenging to diagnose using conventional imaging. Because it can resemble an unidentified ovarian condition, diagnosing cystic degeneration of subserosal fibroids can be very difficult [7,8,9,10] (Farris et al., 2019; Walker et al., 2020; Anyanwu et. al., 2019; Han et al., 2013).

Ultrasonography (USG) serves as the primary tool for diagnosing suspected uterine leiomyomas [11] (Caruso et al., 2022). The most commonly used technique, transabdominal ultrasonography (TAS), offers a comprehensive view of the pelvic structures; however, it may not be sensitive enough to identify posterior or small fibroids. In contrast, transvaginal ultrasonography (TVS) provides greater resolution and diagnostic accuracy for uterine leiomyomas, particularly small ones [12] (American Institute of Ultrasound in Medicine, 2020). USG typically reveals a hypoechoic or heterogeneous uterine mass. The texture may vary depending on the ratio of fibrous tissue to smooth muscle and any degenerative changes present [11,13] (Caruso et al., 2022; Woźniak and Woźniak, 2017). Despite these advantages, ultrasound has notable limitations. Its diagnostic accuracy may be reduced in patients with a high body mass index or when fibroids are located deep within the pelvis. Moreover, while several studies have focused on the number, size, and location of fibroids using ultrasound, limited research has addressed their capacity to detect and characterize degenerative changes, such as cystic or calcific degeneration [14] (Palheta et al., 2023).

MRI is generally considered the gold standard for fibroid evaluation due to its superior soft tissue contrast and ability to detect various types of degeneration [15] (Vanderhoff et al., 2022). However, it is associated with high costs, longer scan times, and contraindications in patients with certain implants or metallic devices [16] (Center for Devices and Radiological Health, 2017). MRI becomes crucial when USG results are inconclusive because it offers superior diagnostic accuracy and provides detailed information about fibroid characteristics and related uterine pathologies [17,18] (Kaushik et al., 2008; Raffone et al., 2024).

In ultrasonography, the leiomyoma degeneration causes a varied and frequently heterogeneous appearance, with minimal or irregular enhancement. Cystic degeneration can manifest as irregular anechoic areas, while calcific degeneration often produces high-level echoes accompanied by distal acoustic shadowing. Gadolinium-enhanced MRI effectively differentiates between degenerated and non-degenerated leiomyomas. Cystic leiomyomas exhibit decreased T1 and increased T2 signal intensities. Calcification appears as low to intermediate signal intensity [6,17,19] (Kaushik et al., 2008; McLucas, 2008; El-Feky & Farooq, 2010).

Degenerative changes in fibroids present as acute abdominal or pelvic discomfort and, in some cases, fever [8] (Farris et al., 2019). They can negatively impact female fertility and often cause pain and discomfort. Fibroids may affect fertility by altering the shape of the uterus, obstructing the fallopian tubes, or interfering with embryo implantation [20] (Leary, 2023). While ultrasound is commonly used to diagnose and monitor uterine leiomyomas, there is limited research on evaluating degenerative changes in these tumors using ultrasound imaging. Most studies emphasize size, location, and number, but overlook specific features like calcification and cystic degeneration. There is a need for more research that applies advanced imaging analysis to better identify and differentiate these changes.

Accurate diagnosis differentiates fibroids from other conditions such as adenomyosis or cancer, predicts responses to therapy, and guides surgical decisions [21] (Lakabi et al., 2025). Precise identification of degenerative changes in uterine myomas is essential for determining an accurate prognosis and planning effective future treatments. For instance, degenerated leiomyomas are contraindicated for embolization because they often respond poorly, having frequently experienced hemorrhagic necrosis [9] (Han et al., 2013). Medical management depends on several factors, including the patient’s age, comorbidities, reproductive goals, fertility preservation needs, symptom severity, suspicion of malignancy, and proximity to menopause [22] (Centini et al., 2024). Asymptomatic cases are monitored clinically. Symptomatic patients are treated based on whether they wish to preserve fertility, keep the uterus, or have no preference, with options ranging from medical therapy to procedures such as uterine artery embolization, focused ultrasound, myomectomy, or hysterectomy. Postmenopausal patients require further evaluation with imaging or biopsy before choosing an appropriate surgical approach [23] (De La Cruz and Buchanan, 2017).

Post-processing methods can enhance biological research by improving image resolution and specificity. Fiji (a distribution of ImageJ) offers several advantages over standard ImageJ, making it a preferred tool for scientific image analysis. As an open-source platform, Fiji is widely used for visualizing, inspecting, quantifying, and validating complex scientific image data [24,25] (Rueden et al., 2017; Schroeder et al., 2020). Through features like automated segmentation, morphological analysis, and quantification tools, ImageJ enables detailed assessments of fibroid characteristics and treatment responses [26] (Medve et al., 2024). Due to its powerful image processing and quantification capabilities, ImageJ’s ability to quantify lesion volume and assess morphological changes could potentially be adapted for ultrasound images [27] (Brito et al. 2022).

While degenerative changes in uterine fibroids negatively impact female fertility, MRI is performed to evaluate fibroid degeneration if ultrasound results are inconclusive. However, MRI is a time-consuming and expensive modality that may not be readily accessible in all healthcare settings, particularly in low-resource regions. Given these limitations, there is an urgent need for reliable, accessible, and cost-effective post-imaging analysis that can assist in assessing degenerative changes. The study aims to explore alternative methods that could complement or partially substitute MRI, improving diagnostic accessibility and efficiency in evaluating fibroid degeneration. It utilizes MRI as the gold standard to evaluate the accuracy of using Fiji for assessing degenerative changes in fibroids.

## 2. Methods and Procedures

### 2.1. Research Design

This analytical cross-sectional study with comparative components collected retrospective cases documented between 2020 and 2024 and incorporated both quantitative and qualitative elements. The main objective was to evaluate the effectiveness of post-processing sonographic techniques for detecting degenerative changes in leiomyomas. Specifically, the study analyzed fibroids in ultrasound images using *Fiji (ImageJ) version 20250529-2217* by identifying qualitative parameters such as the presence of degenerative changes (e.g., cystic changes, calcification) and echotexture, as well as quantitative image metrics including mean intensity, standard deviation, minimum, maximum, skewness, solidity, and kurtosis. These parameters were then compared with routine ultrasound and MRI findings. Furthermore, the study aimed to calculate the accuracy, sensitivity, specificity, and positive and negative predictive values of ultrasound with post-processing techniques, using MRI as the gold standard.

### 2.2. Research Settings

The study was conducted at King Saud Medical City (KSMC). Data collection took place from November 2024 to April 2025.

### 2.3. Target Population, Sample Size, Inclusion, and Exclusion Criteria

The study targeted individuals diagnosed with uterine leiomyomas, evaluated using both sonography and MRI. Inclusion criteria were females of childbearing age (18–50 years) with available medical records and a confirmed diagnosis of leiomyomas based on both ultrasound and MRI, while exclusion criteria included patients who had undergone only one imaging procedure (either ultrasound or MRI) and pregnant women with leiomyomas. A purposive sampling technique was applied, and the study included 41 cases of leiomyomas divided into two groups: fibroids with and without degenerative changes. Data on patients with degenerative changes were collected over the past five years from KSMC, identifying 21 cases based on MRI; however, two were excluded due to pregnancy, leaving 19 cases with degenerative changes. Additionally, 22 cases without degenerative changes were included for comparison.

### 2.4. Research Method: Data Collection and Image Analysis Using Fiji

The data for this study, including ultrasound images, ultrasound reports, and MRI reports, were collected retrospectively by reviewing existing medical records and accessing imaging data through hospitals’ Picture Archiving and Communication System (PACS). The ultrasound images were analyzed using Fiji. A structured data collection sheet was used to systematically record clinical and imaging findings, ensuring consistency and accuracy during analysis.

**Figure 1:**
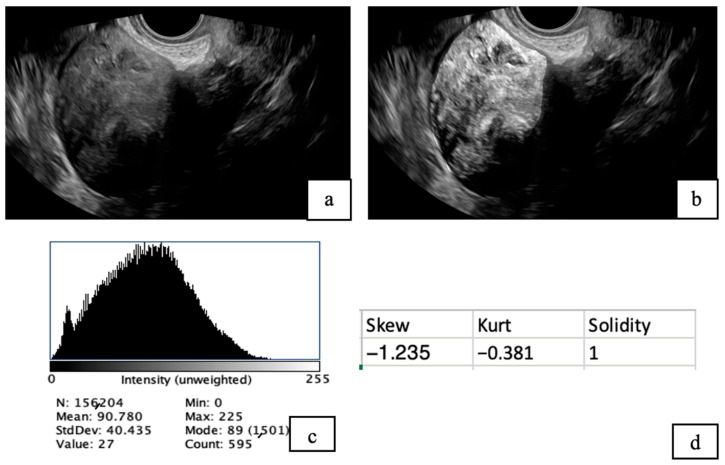
TVS images of uterine fibroids before (**a**) and after analysis using Fiji software (**b**). The post-analysis image highlights the presence of tiny cystic areas within the fibroid. A histogram (**c**) was generated to evaluate pixel intensity distribution, and additional quantitative analysis (**d**) was conducted to assess tissue characteristics, including skewness, solidity, and kurtosis.

Ultrasound images and reports:
All patients were evaluated with pelvic ultrasonography (TAS and TVS). Both images and reports were collected. The parameters obtained from the reports include patient age, symptoms, fibroid location, echotexture, presence of degenerative changes, and type of changes.
MRI reports:
All patients underwent multiplanar, multisequence pelvic MRI with intravenous contrast enhancement to provide a detailed evaluation of uterine fibroids and their internal characteristics. The parameters obtained from the reports include location, presence of degenerative changes, and type of these changes. The radiologist who reported MRI did not know the result of the ImageJ sonographic reports.
Fiji ImageJ Analysis:
Fibroid ultrasound images were analyzed using *Fiji (ImageJ) version 20250529-2217* [28] (Schindelin et al., 2012) following a standardized protocol. Images were first opened in Fiji and converted to 8-bit grayscale for uniformity. Contrast was adjusted to optimize visibility of the fibroid structures. Segmentation was performed by manually delineating regions of interest (ROIs) encompassing the fibroids using selection tools. A histogram was utilized to obtain mean intensity, standard deviation (SD), minimum, and maximum values. Other quantitative measurements were obtained, including skewness, solidity, and kurtosis (Figure 1).
In ImageJ, intensity refers to the brightness level of each pixel in a grayscale image, typically measured on a scale from 0 (black) to 255 (white) in 8-bit images.
Skewness measures how much a data distribution is asymmetrical around its mean. If skewness is zero, the data is perfectly balanced. Positive skewness means the distribution has a longer tail on the right side, with some unusually high values. Negative skewness means a longer tail on the left, with some unusually low values.
Solidity is a shape descriptor that measures how much an object fills its convex boundary. A solidity close to 1 means the shape is solid and compact without many indentations, while lower values indicate a more irregular or fragmented shape.
Kurtosis describes how much intensity has extreme values compared to a normal distribution. Based on interpretation, it indicates whether the data has a sharp peak with heavy tails (high kurtosis > 3), a flat shape with light tails (low kurtosis < 3), or a normal, bell-shaped distribution (moderate kurtosis = 3).

### 2.5. Statistical Analysis

Descriptive statistics, including means, standard deviations, and frequency distributions, were used to summarize demographic and imaging characteristics. Crosstabulation analysis with chi-square testing was performed to compare categorical variables between imaging modalities. Diagnostic accuracy metrics such as sensitivity, specificity, positive predictive value, negative predictive value, and overall accuracy were calculated to assess performance. Receiver operating characteristic (ROC) curve analysis was conducted to evaluate the discriminatory ability of each imaging method, with the area under the curve (AUC) serving as a key comparative measure. An independent samples *t*-test was conducted to assess differences in Fiji parameters between fibroids with and without degenerative changes. All statistical analyses were performed using SPSS V 29 software, with *p*-values < 0.05 considered statistically significant.

### 2.6. Ethical Considerations

The study received approval from the Institutional Review Board (IRB) at Princess Nourah bint Abdulrahman University (IRB No: 25-0028/Date: 28 January 2025) and formal approvals from King Saud Medical City (H1RI-19-Feb25-06/Date: 24 February 2025). Patient confidentiality and data security were prioritized to ensure anonymity by removing identifiable information from ultrasound images before analysis. Since the research involved pre-existing data and did not require direct patient interaction, informed consent was unnecessary.

## 3. Results

### 3.1. Patient Demographics

**Figure 2:**
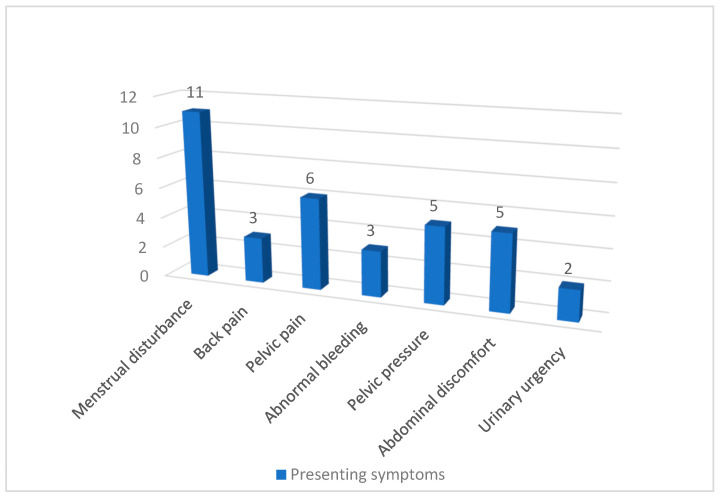
Chief Complaints of Patients with Fibroids.

A total of 41 patients were included in the study. The age range of participants was 31 to 56 years, with a mean age of 44.27 ± 6.66 years. The most common chief complaints were menstrual disturbance (26.8%), pelvic pain (14.6%), pelvic pressure (12.2%), abdominal discomfort (12.2%), and back pain (7.3%), among others (Figure 2).

### 3.2. Ultrasound Findings

Ultrasound imaging revealed degenerative changes in 70.7% of cases, with cystic degeneration present in all of them. Most fibroids were intramural (75.6%), while subserosal fibroids accounted for 19.5% (Table 1). The mean fibroid volume measured by ultrasound was 144.48 ± 153.73 cm^3^ (Table 2).

### 3.3. Postprocessing Findings (Fiji ImageJ)

After postprocessing using ImageJ, fibroid images exhibited a heterogeneous echotexture in 97.6% of cases (Table 3), with degenerative changes identified in 48.8%. Notably, cystic degeneration was present in 46.3%, while calcification was observed in 17.1% of cases. Statistical features extracted from ImageJ analysis indicated a mean intensity of 78.85 ± 26.81. The skewness of the intensity distribution was −1.47 ± 0.99, indicating a negatively skewed histogram. Most areas appear brighter, while fewer regions contain darker pixels.

The solidity of the fibroid regions was 0.99 ± 0.013, indicating that the segmented fibroid areas were nearly solid with minimal concavity or internal irregularity. A solidity value close to 1 reflects compact and well-defined lesion boundaries.

The kurtosis of the intensity distribution was 1.539 ± 2.72, indicating pixel intensities are closer to the average, and extreme bright or dark areas are less common (Table 2).

### 3.4. MRI Findings

MRI confirmed degenerative changes in 46.3% of fibroids (Table 4), consistent with postprocessing results. Most fibroids were located intramurally (63.4%), and 90.2% of MRI scans utilized contrast-enhanced protocols. Cystic degeneration was the most common type observed.

### 3.5. Comparison of Ultrasound and Fiji ImageJ with MRI

A crosstabulation analysis comparing ultrasound and postprocessed ultrasound using ImageJ against MRI findings demonstrated distinct differences in diagnostic performance (Table 5):

- Ultrasound detected degenerative changes in 70.7% of cases, while MRI confirmed changes in 46.3% of cases (*p* = 0.322).
- ImageJ processing identified degenerative changes in 48.8%, which had a statistically significant association with MRI findings (*p* < 0.001).

**Table 5:** Crosstabulation Analysis Comparing Ultrasound and Postprocessed Ultrasound (ImageJ) to MRI Findings.

		Degenerative Changes in MRI		Total	*p*-Value
		No	Yes		
Degenerative changes in US	No	5	7	12	0.322
	Yes	17	12	29	
Degenerative changes in Fiji ImageJ-US	No	18	3	21	<0.001 *
	Yes	4	16	20	
Total		22	19	41	

* Significant association.

## 4. Diagnostic Accuracy Metrics

ImageJ outperformed ultrasound across all diagnostic metrics, showing higher sensitivity (84.2% vs. 63.2%) and specificity (81.8% vs. 22.7%). Postprocessing significantly improved accuracy, increasing correct classifications to 82.9% compared to 41.5% for standard ultrasound. The stronger positive predictive value (80.0%) and negative predictive value (85.7%) of ImageJ suggest improved reliability in identifying degenerative changes (Table 6).

**Figure 3:**
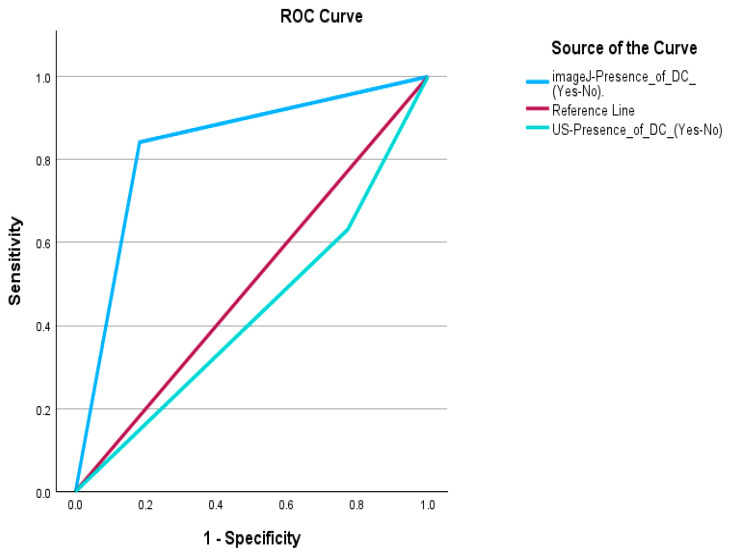
Receiver Operating Characteristic (ROC) Curve for Ultrasound and Postprocessed Ultrasound Using ImageJ Compared to MRI.

The ROC curve analysis compares the diagnostic accuracy of standard ultrasound (US) and postprocessed ultrasound using ImageJ for detecting degenerative changes in fibroids, with MRI as the reference standard (Figure 3). The area under the curve (AUC) for US is 0.429, indicating poor diagnostic performance, while ImageJ achieves a significantly higher AUC of 0.830, demonstrating strong accuracy in distinguishing positive and negative cases. Statistically, US shows no significant discrimination capability (*p* = 0.438), whereas ImageJ exhibits a high correlation with MRI findings (*p* < 0.001), highlighting its superior effectiveness in identifying degenerative changes.

## 5. Discussion

This study aimed to evaluate the diagnostic performance of post-processed ultrasound imaging using ImageJ software for detecting fibroid degeneration, with MRI as the reference standard. The study employed conventional ultrasound for initial imaging, followed by Fiji/ImageJ postprocessing, and MRI for validation. Accurate identification of fibroid degeneration is clinically significant, as it impacts management decisions, surgical planning, and symptom correlation.

Data were collected from 41 patients with a mean age of 44.27 ± 6.66 years. This age group corresponds to the reproductive and perimenopausal phases, during which elevated levels of estrogen and progesterone are known to stimulate fibroid growth. These hormonal effects have been well documented in the recent literature, where progesterone and estrogen were found to promote fibroid growth through cellular proliferation and extracellular matrix production [29] (Ali et al., 2023). The most commonly reported symptom was menstrual disturbances (26.8%), followed by pelvic pain (14.6%), consistent with previously published reports linking fibroid-related symptom burden to size and degeneration type [30] (Vannuccini et al., 2022).

Most of the fibroids identified were classified as intramural as fibroids are developed within the myometrium and then grow either towards the endometrium (submucosal or towards the serosal layer (subserosal). The mean volume of them is considered large, which is often symptomatic; it may distort the uterine contour [31] (Stewart et al., 2017).

The study demonstrated that standard ultrasound identified degenerative changes in 70.7% of cases, whereas postprocessed ultrasound using ImageJ detected changes in 48.8%, closely matching the 46.3% detection rate by MRI. Importantly, only ImageJ findings showed a statistically significant association with MRI (*p* < 0.001), in contrast to conventional ultrasound (*p* = 0.322). These results highlight the added value of postprocessing techniques in enhancing image interpretation accuracy and reliability. ImageJ achieved higher sensitivity (84.2%), specificity (81.8%), and overall accuracy (82.9%) compared to standard ultrasound (sensitivity 63.2%, specificity 22.7%, accuracy 41.5%). These diagnostic improvements align with the study objective and support the alternative hypothesis that postprocessed ultrasound enhances the detection of degenerative changes in leiomyomas.

Our findings are consistent with previous literature that acknowledged the limitations of conventional ultrasound in accurately characterizing degenerative changes in fibroids. Brown et al. (2011) and Han et al. (2013) [5,9] emphasized the challenges in distinguishing between different types of degeneration, particularly cystic and hyaline forms, due to their variable echotexture and signal intensity. Ultrasound’s heterogeneous imaging of fibroids was observed in 85.4% of cases in this study, which reflects the variability in echogenicity associated with varying degrees of degeneration, fibrosis, or calcification within the fibroid tissue. This heterogeneity can influence both diagnosis and treatment planning [10] (Walker et al., 2020).

MRI, regarded as the gold standard for fibroid characterization [15] (Vanderhoff et al., 2022), confirmed cystic degeneration in 46.3% of cases, a figure that matched both ImageJ and standard ultrasound findings. However, postprocessed ultrasound achieved a far superior correlation with MRI findings, as demonstrated by the ROC analysis where ImageJ had an AUC of 0.830 compared to 0.429 for conventional ultrasound. This supports the literature [25,26] (Schroeder et al., 2020; Medve et al., 2024) advocating for the integration of advanced image processing techniques like ImageJ in routine diagnostics to enhance reproducibility and minimize observer variability.

## 6. Implications of the Study

This study offers meaningful implications for clinical practice and future research. First, the findings demonstrate the potential of ultrasound image postprocessing, specifically through ImageJ, to significantly improve the diagnostic accuracy of fibroid degeneration. This has considerable relevance for resource-limited healthcare settings where MRI may not be readily available or accessible. By enabling more accurate detection of degenerative changes in leiomyomas, postprocessing could support better-informed clinical decisions, appropriate triaging, and planning of surgical or conservative management.

Second, the use of ImageJ introduces a reproducible, transparent, and low-cost tool for image analysis, promoting greater diagnostic consistency. This is particularly valuable in low-to-middle-income countries where variability in ultrasound interpretation and limited radiological expertise often hinder effective gynecological care. Furthermore, incorporating postprocessed image analysis into training curricula may enhance the skill set of sonographers and radiologists, bridging the gap between conventional imaging and high-end diagnostic tools.

Additionally, the high correlation between postprocessed ultrasound and MRI supports the role of ImageJ-enhanced ultrasound as a viable surrogate when MRI is contraindicated or unavailable. It may also serve as a monitoring tool for patients with known fibroids, helping to identify changes in echotexture or morphology suggestive of degeneration without repeated MRI exposure. These benefits could ultimately reduce diagnostic delays, unnecessary referrals, and healthcare costs.

## 7. Study Limitations

Despite the promising findings, several limitations must be acknowledged. First, the sample size was relatively small (*n* = 41), limiting the statistical power of subgroup analyses and generalizability of results. This may also explain the higher-than-expected prevalence of cystic degeneration, which contrasts with previous reports such as Han et al. (2013) [9]. A larger, multicenter study would be valuable to validate these findings across diverse populations.

Second, the retrospective design carries inherent limitations, including selection bias and incomplete documentation.

Third, although ImageJ provides robust image quantification capabilities, it remains user-dependent and requires technical proficiency for optimal use. Inconsistent image acquisition, differing probe frequencies, and user variability in capturing ultrasound images may affect postprocessing results. Furthermore, the study did not evaluate inter- and intra-observer variability in the interpretation of ImageJ outputs, which is important for real-world applications.

Lastly, while we explored multiple statistical features extracted from ImageJ, no single metric demonstrated a statistically significant association with degeneration status. Future studies should explore machine learning classifiers or multivariate models incorporating these features to better predict degeneration types.

## 8. Conclusions and Recommendations

This study addressed the challenge of limited diagnostic clarity in conventional ultrasound by utilizing ImageJ (Fiji) software to enhance the evaluation of degenerative changes in leiomyomas. The results confirmed that integrating ultrasound with post-processing image analysis significantly improves the precision and reliability of detecting degenerative features, thereby reducing the need for more expensive or less accessible imaging modalities such as MRI. This aligns with the evolving trend in medical imaging toward adopting open-source, AI-driven tools to enhance diagnostic workflows. The study supports the incorporation of such technologies in routine clinical practice to promote accuracy, efficiency, and broader accessibility in patient care.

Several recommendations are proposed for future research, considering the limitations encountered during this study. Firstly, future studies with a prospective nature should include a larger sample size to enhance the generalizability of findings. It is also advisable to allocate a longer timeframe for data collection, allowing sufficient time to navigate administrative procedures, particularly the delays associated with obtaining hospital approval. Enhancing the completeness and accuracy of patient medical records is crucial, as detailed clinical histories are vital for establishing stronger correlations between clinical symptoms and specific types of fibroid degeneration.

## Figures and Tables

**Table 1:** Distribution of Fibroid Characteristics Identified by Ultrasound Imaging.

Characteristic	Category	N	%
US-Location	Intramural	31	75.6%
	Submucosal	2	4.9%
	Subserosal	8	19.5%
Ultrasound Echotexture	Heterogeneous	35	85.4%
	Homogeneous	6	14.6%
Presence of DC	Yes	29	70.7%
	No	12	29.3%
Cystic Degeneration	Yes	29	70.7%
	No	12	29.3%
Calcification	No	41	100.0%

**Table 2:** Postprocessed Ultrasound Findings Using Fiji ImageJ—Statistical Features of Fibroid Images.

Variable	Minimum	Maximum	Mean	Std. Deviation
Mean Intensity	17.62	143.754	78.84795	26.81
SD	16.49	65.716	41.32661	12.27
Min	0	55	3.93	10.24
Max	113	255	223.83	31.37
Skewness	−3.25	1.135	−1.47	0.99
Solidity	0.91	1.000	0.99	0.013
Kurtosis	−1.45	9.367	1.539	2.47

**Table 3:** Frequency Distribution of Fibroid Characteristics Identified by Fiji ImageJ Analysis.

Characteristic	Category	N	%
ImageJ Echotexture	Heterogeneous	40	97.6%
	Homogeneous	1	2.4%
Presence of DC	Yes	20	48.8%
	No	21	51.2%
Cystic Degeneration	Yes	19	46.3%
	No	22	53.7%
Calcification	Yes	7	17.1%
	No	34	82.9%

**Table 4:** Frequency Distribution of Fibroid Characteristics Identified in MRI Scans.

Characteristic	Category	N	%
MRI Location	Extra Serosal	1	2.4%
	Intramural	26	63.4%
	Submucosal	3	7.3%
	Subserosal	11	26.9%
MRI Protocol	With Contrast	37	90.2%
	Without Contrast	4	9.8%
Presence of DC (MRI)	Yes	19	46.3%
	No	22	53.7%
Degeneration Type	Cystic	19	46.3%
	No	22	53.7%

**Table 6:** Comparison of Diagnostic Performance of Standard Ultrasound (US) vs. Postprocessed Ultrasound Using ImageJ for Detecting Degenerative Changes in Fibroids.

Metric	US	ImageJ
Sensitivity	63.2%	84.2%
Specificity	22.7%	81.8%
Positive Predictive Value (PPV)	41.4%	80.0%
Negative Predictive Value (NPV)	41.7%	85.7%
Accuracy	41.5%	82.9%

## Data Availability

The original contributions presented in this study are included in the article. Further inquiries can be directed to the corresponding author.

## Abbreviations

AUC	Area Under the Curve
IRB	Institutional Review Board
KSMC	King Saud Medical City
MRI	Magnetic Resonance Imaging
NPV	Negative Predictive Value
PACS	Picture Archiving and Communication System
PPV	Positive Predictive Value
ROC	Receiver Operating Characteristic
ROI	Region of Interest
SD	Standard Deviation
TAS	Transabdominal Ultrasonography
TVS	Transvaginal Ultrasonography
US	Ultrasound
USG	Ultrasonography
